# ID 358 - Uso do Geoprocessamento como Tecnologia para Avaliação em Saúde: investigação da tendência espacial da mortalidade por doença de Alzheimer no Brasil

**DOI:** 10.5327/2237-9622.2025.v34s1.358

**Published:** 2025-11-25

**Authors:** Luana Teles de Resende, Caio César Balthazar da Silveira Vidal, Luma Teles de Resende, Rhuan Alexander da Silva Dornelles, Matheus Santos Melo, Lis Campos Ferreira, Breno José Alencar Pires Barbosa

**Keywords:** doença de Alzheimer, mortalidade, análise espacial

## Abstract

**Introdução:**

A doença de Alzheimer (DA) é uma condição neurodegenerativa com uma alta prevalência na população brasileira, especialmente em idosos. Com o aumento da expectativa de vida, a prevalência da DA tende a crescer, tornando a compreensão dos padrões da mortalidade essencial para direcionar políticas de saúde eficazes. O objetivo deste estudo foi analisar os padrões espaciais e espaço-temporais da mortalidade relacionada à doença de Alzheimer no Brasil, por meio do geoprocessamento como tecnologia para avaliação em saúde.

**Método:**

Estudo ecológico de base populacional utilizando dados sobre mortes relacionadas à doença de Alzheimer (CID-10 F00 e G30) do Sistema de Informações sobre Mortalidade (SIM) do Ministério da Saúde do Brasil, de 2006 a 2021. Dados demográficos e populacionais foram obtidos do Instituto Brasileiro de Geografia e Estatística (IBGE). A análise de tendência temporal foi realizada utilizando o método Joinpoint, identificando mudanças nas tendências com o teste de Permutação de Monte Carlo, calculando a Mudança Percentual Anual (APC) e a Mudança Percentual Anual Média (AAPC). Estatísticas de varredura espaço-temporal foram aplicadas para identificar aglomerados de alto risco, utilizando critérios específicos para aglomerados significativos e ferramentas como R, QGis e Joinpoint Regression SoftwareTM.

**Resultados:**

Entre 2006 e 2021, foram registrados 441.509 óbitos de idosos relacionados à DA no Brasil, com 244.480 casos identificados como causa básica. A maioria dos óbitos ocorreu na Região Sudeste (53,54%) e em pessoas com 80 anos ou mais (73,1%). As mulheres apresentaram uma prevalência maior, com 64,22% dos óbitos. A maioria das mortes ocorreu em hospitais (58,73%) e houve aumento significativo de óbitos ao longo dos anos, passando de 2,44% em 2006 para 10,46% em 2021. A análise temporal mostrou um crescimento das taxas de mortalidade por idade e sexo, com maior impacto nas mulheres. Espacialmente, as Regiões Sul e Sudeste tiveram as maiores taxas de mortalidade. Um aglomerado urbano foi identificado entre 2011 e 2021, com 218.826 óbitos, superando as expectativas (111.223 óbitos) e indicando um risco três vezes maior de mortalidade nessa área. O teste de razão de verossimilhança (LLR) apresentou significância estatística (p <0,0001), sugerindo fatores específicos contribuindo para essa mortalidade elevada. A autocorrelação espacial foi positiva (Moran I = 0,5893; p-valor = 0,001), indicando aglomerados. A análise LISA revelou que os municípios de baixo risco estão nas Regiões Norte e Nordeste, enquanto os de alto risco se concentram no Sul e Sudeste. A análise de varredura espaço-temporal identificou um aglomerado de risco significativo (p < 0,05) em uma localização específica.

**Conclusão:**

Os dados mostraram aumento na mortalidade ao longo do tempo, possivelmente devido a melhorias no diagnóstico e no registro da doença. Regiões Sudeste e Sul apresentaram as maiores taxas de mortalidade, indicando fatores socioeconômicos e de saúde que precisam ser investigados. O geoprocessamento revelou aglomerados de alta mortalidade, sugerindo que fatores locais, como poluição e acesso a cuidados, podem influenciar essas disparidades. As limitações incluem subnotificação e variações na qualidade das informações entre regiões. Apesar disso, os resultados fornecem reflexões importantes para entender a distribuição da mortalidade por DA e identificar áreas que necessitam de atenção em saúde pública.

